# The use of *in vitro* bioassays and chemical screening to assess the impact of a minimally processed vegetable facility on wastewater quality

**DOI:** 10.3389/ftox.2024.1439126

**Published:** 2024-09-16

**Authors:** N. H. Aneck-Hahn, M. C. Van Zijl, L. Quinn, C. Swiegelaar, N. Nhlapo, W. de Bruin, L. Korsten

**Affiliations:** ^1^ Environmental Chemical Pollution and Health Research Unit, School of Health Systems and Public Health, Faculty of Health Sciences, University of Pretoria, Pretoria, South Africa; ^2^ Environmental Chemical Pollution and Health Research Unit, Department of Urology, Faculty of Health Sciences, University of Pretoria, Pretoria, South Africa; ^3^ Organic Analysis Laboratory, National Metrology Institute of South Africa, Pretoria, South Africa; ^4^ Department of Plant and Soil Sciences, Faculty of Natural and Agricultural Sciences, University of Pretoria, Pretoria, South Africa

**Keywords:** food-processing facility, endocrine-disrupting chemicals, *in vitro* bioassays, estrogenic activity, disinfectants, pesticides, chemical screening, wastewater

## Abstract

Fruit- and vegetable-processing facilities may contaminate wastewater via contaminants found in the produce and disinfecting chemicals used. These contaminants may include agrochemicals, pesticides, and disinfectants such as chlorine and quaternary ammonium compounds (QACs). Some compounds may exhibit harmful endocrine-disrupting activity. This study investigated the impact of a minimally processed vegetable facility on wastewater quality via *in vitro* bioassays and chemical screening. Estrogen activity was assessed via a yeast estrogen screen (YES), and (anti-)androgenic and glucocorticoid activities were evaluated via an MDA-kb2 reporter gene assay. The samples were screened via gas and liquid chromatography–tandem mass spectrometry (GC-MS/MS and LC-MS/MS) to identify target compounds, and GC coupled with time-of-flight mass spectrometry (GC-TOFMS) was used for non-targeted screening. Sample complexity and chemical profiles were assessed using GC-TOFMS. Estrogenic activity was detected in 16 samples (n = 24) with an upper limit of 595 ± 37 ng/L estradiol equivalents (EEqs). The final wastewater before discharge had an EEq of 0.23 ng/L, which is within the ecological effect-based trigger value range for the estrogenic activity of wastewater (0.2–0.4 ng/L EEq). Androgenic activity was detected in one sample with a dihydrotestosterone equivalent (DHTEq) value of 10 ± 2.7 ng/L. No antiandrogenic activity was detected. The GC-MS/MS and LC-MS/MS results indicated the presence of multiple pesticides, nonylphenols, triclocarban, and triclosan. Many of these compounds exhibit estrogenic activity, which may explain the positive YES assay findings. These findings showed that wastewater from the facility contained detergents, disinfectants, and pesticides and displayed hormonal activity. Food-processing facilities release large volumes of wastewater, which may affect the quality of the water eventually being discharged into the environment. We recommend expanding conventional water quality monitoring efforts to include additional factors like endocrine activity and disinfectant byproducts.

## 1 Introduction

Minimally processed fruits and vegetables are rich in nutrients essential for human health. The industry has experienced rapid growth (Oniciuc et al., 2019), with the South African fruit- and vegetable-processing sector valued at approximately USD 1 million in 2015 (Chitonge, 2021). However, fresh produce can act as a vehicle for foodborne pathogens such as *Salmonella* spp., *Listeria monocytogenes*, and pathogenic *Escherichia coli* (Dewey-Mattia et al., 2018 *)*. According to the World Health Organization, the consumption of unsafe food is responsible for almost 600 million cases of foodborne disease and 420,000 deaths annually (WHO, 2015).

Food-processing facilities commonly use disinfectants like chlorine to wash fruits and vegetables, and quaternary ammonium compounds (QACs) to clean surfaces, thereby minimizing the risk of microbiological contamination (van Dijk et al., 2022). Chlorine-based solutions are commonly used because of their low cost and broad spectrum of biocidal activity (Pedreira et al., 2021). QACs such as triclosan are routinely used for hand sanitation and cleaning contact surfaces such as cutting boards, crates, and utensils. These disinfectants are effective against various microorganisms and are nonirritating and noncorrosive (Melin et al., 2016).

Food-processing facilities use large volumes of water during the washing process, resulting in wastewater that contains disinfection byproducts and contaminants. Chlorine-based disinfectants result in chlorine byproducts, such as chlorites and chlorates. Chlorate in foodstuffs is associated with kidney failure, hinders iron absorption, and affects thyroid function (Garvey, 2019). Moreover, the toxicity of QACs has been reported among aquatic organisms and rodents (Zheng et al., 2021; Hrubec et al., 2017; Zhang et al., 2015).

QACs have also been shown to cause genotoxic effects in mammalian and plant cells at environmentally relevant concentrations (Ferk et al., 2007). Melin et al. (2016) reported that QACs were associated with decreased fertility in mice, and another study revealed neural tube defects in rodents, suggesting that QACs are teratogenic (Hrubec et al., 2017). QACs might reduce rodent fertility through endocrine-disrupting mechanisms, indicating a need to investigate potential toxicity to human reproductive systems (Melin et al., 2016; Hrubec et al., 2017). Additionally, Zheng et al. (2021) reported that QACs bioaccumulate in human blood. Triclosan, used in some hand sanitizers, is also a known endocrine disruptor (Milanović et al., 2023). Most hand hygiene products in South Africa contain endocrine-disrupting chemicals (EDCs) (de Bruin et al., 2024). Endocrine disruptors are associated with reproductive disorders, thyroid-related disorders, neurodevelopmental disorders, hormone-related cancers, adrenal disorders, bone disorders, metabolic disorders, and immune diseases (Bergman et al., 2013). The potential adverse health effects of chronic, low-dose exposure to EDCs are of particular concern (Melin et al., 2016).

Fresh produce can also be contaminated by heavy metals, pesticides, agrochemicals, disinfectants, and other chemicals used in producing, packaging, and transporting food products (Garvey, 2019). Pesticide residues may be introduced from pre- and postharvest applications. Mutengwe et al. (2016) evaluated fruit and vegetable samples from major fresh produce markets in South Africa for pesticide residues and estrogenic activity. More than 30% of the samples contained pesticide residues although only one sample exceeded the maximum residue levels, and 26% showed estrogenic activity (Mutengwe et al., 2016). QAC residues have been detected in fruit and vegetable samples from different countries (Xiang et al., 2015).

The increasing popularity of minimally processed fruits and vegetables reflects a broader societal shift toward health-conscious eating habits. Despite their perceived benefits for human health, little is known about the environmental impacts of the facilities that process these foods, particularly in terms of water pollution. This study aims to fill this gap by investigating the presence of EDCs and other contaminants in the wastewater of a facility specializing in the production of minimally processed vegetables. By investigating the levels and types of pollutants, this research seeks to assess the environmental footprint of such facilities and contribute to the development of more sustainable food-processing practices.

## 2 Materials and methods

Ethical approval for this study was obtained from the Ethics Committee of the University of Pretoria, Faculty of Health Sciences (reference number: 137/2024).

### 2.1 Study site

This study was conducted at a large, temperature-controlled, minimally processed vegetable facility in Gauteng, South Africa. The facility adheres to national food safety regulations and is compliant with Hazard Analysis Critical Control Point-accredited and Food Safety System Certification 2200.

The processing facility draws water from a borehole, which is pretreated with chlorine before being fed into the processing facility. This point was considered the control or reference point. The water is primarily used to wash incoming fresh produce and for general cleaning operations, including floors, walls, drains, equipment, cutting boards, and utensils. The processing facility is deep cleaned daily by cleaning teams during nonoperational hours (at night). All surfaces are disinfected with QAC-based products, while the drains are disinfected with peroxides or chlorine-based compounds. During the day, only control water is used for cleaning, and no additional chemicals are used. The facility discharges wastewater through a drainage outlet into a sewage line that eventually enters a wastewater treatment plant (WWTP) approximately 20 km away. The WWTP is designed to treat 16 ML of industrial and domestic wastewater per day.

### 2.2 Sample description and collection

Water samples were collected from the facility during four sampling events in February 2023 (South African summer), March 2023 (autumn), June 2023 (winter), and September 2023 (spring) to account for seasonal variations. The initial sampling plan included sampling from seven points in the facility, but for practical reasons, samples could not be taken from the drain in low care (sample 5) and were excluded from the project. Full descriptions of the remaining six sampling points are given in Table 1. The samples were collected in 1-L glass bottles (1 L for bioassays and 1 L for chemical analysis at each sample point), transported in cooler boxes with ice packs, and stored at 4°C in the laboratory until extraction (De Jager et al., 2011).

**TABLE 1:** Description of sample points.

Sample point	Sample description
Sample 1	Clean chlorinated water entering the facility (control/reference)
Sample 2	Spent water from high-care basins where utensils and cutting boards are washed
Sample 3	A drain that receives spent water from high-care and low-care basins
Sample 4	Low-care washing baths that are drained
Sample 6	Spent water from low-care basins where utensils and cutting boards are washed
Sample 7	Spent water from low-care baths where crates are washed

Note: There is no sample 5 as it had to be excluded from the project owing to difficulties in sampling from that point.

### 2.3 Bioassay analyses

*In vitro* bioassays are used to assess the biological activity of chemical compounds in water. Reporter gene assays are most commonly used to measure toxicity and estrogenic and androgenic activity. Effect-based methods are used to evaluate water quality and safety, complementing chemical analysis by integrating the effects of known and unknown chemicals and mixture effects (Leusch and Snyder, 2015).

#### 2.3.1 Sample extraction for bioassays

Samples were extracted within 3 days after collection via solid-phase extraction (SPE) using Oasis HLB glass cartridges (Waters, Milford, MA, United States) according to the methods described by De Jager et al. (2011). Before extraction, the samples were filtered through two glass wool filter discs and one 0.45-μm filter to remove vegetable residues. The free chlorine concentration in each sample was measured using MQuant^®^ chlorine test strips from Merck (Darmstadt, Germany). As free chlorine can interfere with the bioassays, 5 mg/L ascorbic acid (Merck, Darmstadt, Germany) was added to the water sample to neutralize 1 mg/L of free chlorine. The pH value of each water sample was adjusted to 3 with 32% hydrochloric acid (Merck, Darmstadt, Germany). The SPE cartridges were preconditioned with 5 mL of ultrapure water and 5 mL of HPLC-grade methanol (Merck, Darmstadt, Germany). The cartridges were equilibrated with 5 mL ultrapure water before loading the samples (1 L/cartridge). The cartridges were dried, wrapped in foil, and stored at −20°C until all the samples were collected and ready for analysis. The extracts were eluted from the cartridges with 5 mL of HPLC-grade methanol (Merck, Darmstadt, Germany), and the solvent was evaporated to dryness under a gentle nitrogen stream. The extracts were reconstituted in 1 mL of HPLC-grade ethanol (Merck, Darmstadt, Germany) and stored at −20°C. The enrichment factor of the extracts was 1,000.

#### 2.3.2 Yeast estrogen screen assay

A yeast estrogen screen (YES) assay was used to analyze the samples for estrogenic activity. The yeast strain was obtained from Xenometrix, Switzerland (Cat. No. N05-230-E). Stock cultures and growth media were prepared, and the YES assay was performed according to the procedure described by De Jager et al. (2011).

The yeast growth medium (5 mL) was inoculated with 50 µL of the ×10 concentrated yeast stock and incubated at 28°C in a rotating water bath at 150–155 revolutions per minute until it became turbid (approximately 24 h). Serial dilutions of the sample extracts and controls were prepared in 96-well microtiter plates (untreated, clear, and flat bottom) in HPLC-grade ethanol (Merck, Darmstadt, Germany). From the dilution plate, 10 µL aliquots were transferred to 96-well assay plates and evaporated to dryness. The aliquots (200 µL) of the assay medium containing the yeast and chromogenic substrate (CPRG, Roche Diagnostics, Mannheim, Germany) were dispensed into each well. Each plate contained at least one row of blanks (assay medium and solvent ethanol) and a standard curve for the positive control 17β-estradiol (E2, Cat. No. E8875, Sigma-Aldrich, St. Louis, MO, United States), ranging from 1 × 10^−8^ M to 1.2 × 10^−15^ M (2.7 × 10^−6^ g/L to 3.2 × 10^−13^ g/L). The plates were sealed with autoclave tape and placed in a naturally ventilated incubator at 29°C for 3–5 days. After 3 days of incubation, the color development of the medium was checked for 3 days (days 3–5) at an absorbance (abs) of 540 nm for color change and 620 nm for turbidity of the yeast culture. The absorbance was measured using a Multiskan Spectrum 96-well plate reader to obtain the best contrast. All the experiments were performed in triplicate. The following equation was applied to correct for turbidity:

$$\text{Corrected value}=\text{test abs }\left(540\text{ nm}\right)\;‐\;\left[\text{test abs }\left(620\text{ nm}\right)\;‐\text{ median blank abs }\left(620\text{ nm}\right)\right].$$

The E2 logarithmic dose–response curve was fitted (sigmoidal function, variable slope) using GraphPad Prism (version 9.5.1), which calculates the minimum, maximum, slope, and EC_50_ values (the concentration where the activity is 50% of the maximum activity obtained by E2) and 95% confidence limits. The detection limit (DL) of the yeast assay was calculated as the absorbance elicited by the solvent control (blank) plus three times the standard deviation. The limit of quantification (LOQ) is equivalent to the EC_10_ value of the E2 standard curve (i.e., the concentration where the activity is 10% of the maximum activity obtained by E2). Cytotoxicity was indicated if the absorbance of a well was less than the absorbance elicited by the solvent control (blank) minus three times the standard deviation. The EEqs of samples above the LOQ were interpolated from the E2 standard curve (at EC_50_) and corrected with the appropriate dilution factor for each sample. Equivalent values are reported as the average EEq ± standard deviation.

#### 2.3.3 MDA-kb2 reporter gene assay

The MDA-kb2 reporter gene assay was used to determine (anti-)androgenic activity and glucocorticoid activity, according to the methods described by Wilson et al. (2002). The MDA-kb2 cell line was derived from the human MDA-MB-453 breast cancer cell line via stable transfection with the mouse mammary tumor virus (MMTV) luciferase-neo reporter gene construct. Since both the androgen and glucocorticoid receptors are present in the cell line and act through the MMTV promotor, this assay can assess agonist and antagonist activity for both receptors (Wilson et al., 2002). MDA-kb2 cells (Cat No. CRL-2713) were obtained from the ATCC (Manassas, VA, United States) and maintained in Leibovitz’s L-15 growth media (Gibco, Life Technologies Corporation, Paisley, United Kingdom) supplemented with 10% characterized fetal bovine serum (HyClone Laboratories, Logan, UT, United States). HPLC-grade ethanol (Merck, Darmstadt, Germany) was used to prepare a stock concentration of 10 mM of dihydrotestosterone (DHT; Cat. No. A2570-000; Steraloids, Newport, RI, United States) and a stock concentration of 100 mM of flutamide (Cat. No. F9397; Sigma-Aldrich, St. Louis, MO, United States). The stock solutions were stored in amber glass bottles at −20°C. The reaction buffer (pH 7.8) was prepared in ultrapure water and consisted of 25 mM glycylglycine, 15 mM MgCl_2_, 5 mM ATP, and 0.1 mg/mL BSA. All reaction buffer components were obtained from Sigma-Aldrich (St. Louis, MO, United States). The reaction buffer was stored in 15-mL aliquots at 4°C. A 1 mM luciferin solution was prepared in ultrapure water and stored in 10-mL aliquots at −80°C. Luciferin was purchased from Promega (Madison, Wisconsin, United States).

The cells were seeded at 5 × 10^4^ cells per well in 96-well luminometer plates and allowed to attach overnight. Dosing dilutions were prepared in Leibovitz’s L-15 growth medium containing 10% FBS. Each plate contained an agonist positive control (DHT), a negative control (vehicle only), an antagonist control (DHT plus flutamide), and a background control (vehicle plus flutamide). The DHT standard curve on each plate ranged from 0.0003 nM to 1 nM. Each sample was tested alone or in the presence of 1 nM DHT (to test for antiandrogenic activity) or 10 μM flutamide. To distinguish between androgenic activity and glucocorticoid activity, the samples are co-incubated with flutamide (flutamide suppresses androgenic activity but not glucocorticoid activity). The cells were exposed for 24 h to 100 μL of dosing solution per well at 37°C. After incubation, the cells were washed with phosphate-buffered saline at room temperature and lysed with 25 μL lysis buffer. Luciferase activity was determined using a luminometer and quantified as relative light units. Each well received 25 μL of reaction buffer and 25 μL of 1 mM D-luciferin 5 s later.

The DHT logarithmic dose–response standard curve (sigmoidal function, variable slope) was fitted using GraphPad Prism (version 9.5.1), which calculates the minimum, maximum, slope, and EC_50_ values and 95% confidence limits. The DL of the assay was calculated as the relative light unit value elicited by the solvent control (blank) plus three times the standard deviation. The LOQ is equivalent to the EC_10_ value of the DHT standard curve. The DHT equivalents (DHTEq) of samples above the LOQ were interpolated from the DHT standard curve (at the EC_50_) and corrected with the appropriate dilution factor for each sample. Equivalent values were reported as the average DHTEq assessed on three plates ± standard deviation.

### 2.4 Chemical analysis

All chemical analyses were performed at the National Metrology Institute of South Africa. The method was initially developed and optimized for analyzing pesticides and pesticide metabolites in drinking water and has been adapted to include nonylphenol (a mixture of isomers), triclocarban, and triclosan. The method was developed via analytical standards procured from ISO 17034-accredited producers, Dr. Ehrenstorfer (Augsburg, Germany), Sigma-Aldrich/Fluka (Zwijndrecht, Netherlands), and Restek (Pennsylvania, United States). Stock solutions of 0.5 mg/g were prepared for individual standards in methanol and diluted to 50 ug/g. Working standard mixtures containing all the pesticides analyzed via gas and liquid chromatography–tandem mass spectrometry (GC-MS/MS and LC-MS/MS) were prepared in acetonitrile at concentrations of 3.50 μg/g, 0.25 μg/g, and 0.05 μg/g. The working standards were used to prepare calibration solutions for LC-MS/MS and GC-MS/MS at concentrations of 0.001, 0.005, 0.01, 0.02, 0.05, 0.08, 0.1, 0.2, and 0.3 μg/g. All the solvents were pesticide analysis grade or higher (Burdick and Jackson^TM^, high-purity solvents; Seelze, Germany). Before use, all equipment was cleaned using multiple solvent rinses to minimize the risk of cross-contamination.

#### 2.4.1 Sample extraction for chemical analysis

Initially, water samples (350–500 mL) were extracted using C18 solid-phase extraction disks and eluted with dichloromethane (DCM)/ethyl acetate (3/1; v/v) using an automated water extraction system. This is the validated pesticide extraction method used to analyze drinking water. However, this method cannot be used to analyze samples that contain high levels of organic matter (plant material and fungal growth) and surfactants. Liquid–liquid extraction with a differential freezing approach was performed for samples that could not be extracted via the automated system. Differential freezing allows the water component to be efficiently separated from the DCM/ethyl acetate solvent during extraction. Liquid–liquid extraction is nonspecific, and the extract is intrinsically expected to contain more organic compounds than SPE. The extraction methods used for specific samples are given in Table 2.

**TABLE 2:** Extraction methods used for individual samples.

Sampling date	Sample 1	Sample 2	Sample 3	Sample 4	Sample 6	Sample 7
February 2023	SPE	LLE	SPE	SPE	LLE	SPE
March 2023	SPE	SPE/LLE	SPE	LLE	SPE	SPE
June 2023	SPE	SPE	SPE	SPE	SPE	SPE
September 2023	SPE	LLE	SPE	SPE	SPE	LLE

SPE, automated solid-phase extraction using C18 disks; LLE, liquid–liquid extraction with differential freezing.

#### 2.4.2 Nonpolar organic compound screening—gas chromatography coupled with time-of-flight mass spectrometry

Instrument analysis was performed using a LECO Pegasus 4D Gas Chromatograph coupled with a time-of-flight mass spectrometer. The column used was an Rxi^®^-XLB (30 m, 0.25 mm ID, 0.25 µm df) with a gradient temperature program and a pulsed splitless injection. The oven gradient was optimized to efficiently separate more than 200 pesticides from multiple chemical classes, including nonylphenol, triclosan, and triclocarban.

Total ion chromatograms (TICs) were generated for each of the samples. For the generation of peak tables, gas chromatography coupled with time-of-flight mass spectrometry (GC-TOFMS) was used to detect any organic chemicals present in sufficient concentrations and intermediate to nonpolar. Only chemicals that could be identified via the NIST/EPA/NIH Spectral Library with a match greater than 80% were reported. Spiked water samples enriched at 0.05 μg/g were used for quality control.

#### 2.4.3 Gas chromatography coupled with triple quadrupole mass spectrometry

An Agilent 7010B Triple Quadrupole Mass Spectrometer was used to screen for 287 chemicals, including surfactants, disinfectants, pesticides, and their metabolites. The column used was a Zebron™ ZB-XLB-HT Inferno™ (30 m, 0.25 mm ID, 0.25 µm df). The MS method involves two transitions for each analyte. For a compound to be positively identified, the retention time and the ratio between the two transitions needed to match those of authentic standards enriched into a water sample and extracted via the same method described above.

#### 2.4.4 Liquid chromatography coupled with triple quadrupole mass spectrometry

The samples were screened for 298 chemicals, including surfactants, disinfectants, pesticides, and their metabolites. The chromatographic system used was a Waters ACQUITY Ultra High-Performance Liquid Chromatograph coupled with a TQS mass spectrometer. The column used was an ACQUITY™ HSS T3 C18 (1.8 µm, 2.1 mm ID × 100 mm). The instrumental analysis used was adapted and optimized from the literature (Waters Application note: 720004789). The MS method involves two transitions for each analyte. The same quality control procedures for positive identification were followed as those described for the GC-MS/MS analysis.

## 3 Results

### 3.1 Bioassay results

#### 3.1.1 Yeast estrogen screen assay

The YES assay findings are summarized in Table 3. Sixteen samples were above the DL of the assay, 13 of which could be quantified. Sample 1 served as the control and was below the DL for all the sampling events. Estrogenic activity was detected at all the other sample points for at least two sampling events. Sample 2 was the only sample where estrogenic activity could be quantified for all sampling events.

**TABLE 3:** Estrogenic activity of water samples using the YES assay. The results are expressed as the average EEq value in ng/L ± standard deviation.

Sample point	EEq (ng/L)			
	February	March	June	September
Sample 1	<DL	<DL	<DL	<DL
Sample 2	84 ± 6.9^a^	119 ± 19^a^	294 ± 14^a^	3.0 ± 0.14^a^
Sample 3	<LOQ	0.23 ± 0.01^a^	0.23 ± 0.02^a^	<DL
Sample 4	<LOQ	362 ± 103^a^	15 ± 0.67^a^	11 ± 2.8^a^
Sample 6	595 ± 37^a^	0.13 ± 0.02	<LOQ	<DL^a^
Sample 7	0.13 ± 0.02	<DL	123 ± 3.8^a^	<DL^a^

<DL, below the detection limit of the assay; <LOQ, above the detection limit of the assay but below the limit of quantification; nd, not detected.

^a^ Cytotoxicity.

Cytotoxicity was observed in 13 samples, with sample 7 collected in September 2023 showing the highest cytotoxicity (toxicity was observed even when the sample was diluted ×30). Some of the samples also displayed leaching/creeping activity: sample 2 (February, March, June, and September); sample 4 (March, June, and September); sample 6 (February); and sample 7 (June). These samples all showed toxicity at the highest concentrations, followed by estrogenic activity in the diluted wells. Wells adjacent to the wells displaying toxicity showed estrogenic activity, suggesting leaching of an estrogenic chemical from the sample across the plate. To resolve this problem and prevent false positive results for samples adjacent to samples containing leaching chemicals, no assay medium was added to the wells between samples, followed by a row of blanks to confirm that the samples did not leach across the empty wells.

#### 3.1.2 MDA-kb2 reporter gene assay

Only sample 7, collected in March, tested positive for androgenic activity, with a DHTEq value of 10 ± 2.7 ng/L. None of the samples showed antiandrogenic or glucocorticoid activity, and 10 samples showed cytotoxicity.

### 3.2 Chemical analyses

#### 3.2.1 Chemical screening

**FIGURE 1:**
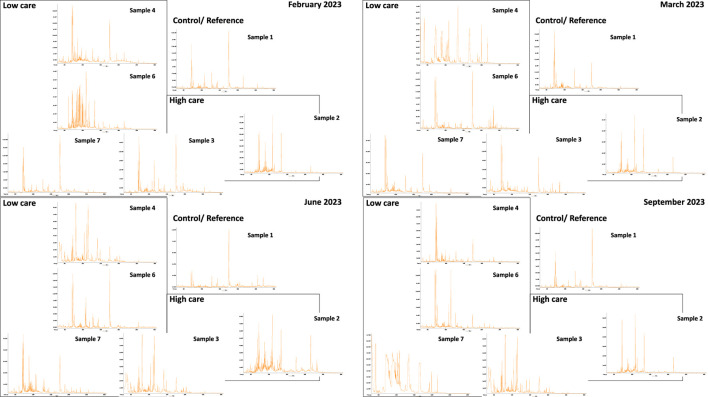
TICs of the various samples in relation to the collection point.

The total ion chromatograms of the various sampling points are shown in Figure 1. Each insert represents a different sampling campaign. Each peak represents a distinct chemical, and more peaks indicate greater complexity (more detectable compounds). The prominent peak in the middle of the total ion chromatogram is an artifact of SPE. It is present in all samples and can be discarded when interpreting results. From the chromatograms, the clean water entering the facility was less complex than the water sampled. The drains were slightly less complex than the basins.

GC-TOFMS revealed that both chorine-based compounds and QACs were present. Notably, QACs were not readily detected and analyzed via gas chromatography. The profiles of these chemical classes were visually investigated using common fragment ions associated with the mass spectra generated by these chemicals, as reported in the literature (McLafferty and Turečk, 1993; Nishikawa et al., 2003), as well as the use of mass spectra for representative analytes (NIST Mass Spectral Search Program for the NIST/EPA/NIH Mass Spectral Library, 2014). For chlorine-based disinfectants, the mass–charge ratios used were 51, 52, 53, 55, 57, 59, 60, 67, 69, 71, 83, 85, 86, 88, 90, 91, 92, 93, 125, 127, 152, 154, 273, 304, 307, and 332 m/z. For QACs, the ratios were 50, 116, 212, 213, 319, 320, 411, and 412 m/z. The extracted ion chromatograms of the various samples revealed a very complex profile for chlorinated compounds, with the highest complexity found in samples 2 and 3. For the QACs, less complexity was identified, likely because they are less amenable to GC analysis. The greatest complexity was found in samples 2 and 4.

#### 3.2.2 LC-MS/MS and GC-MS/MS screening results

**FIGURE 2:**
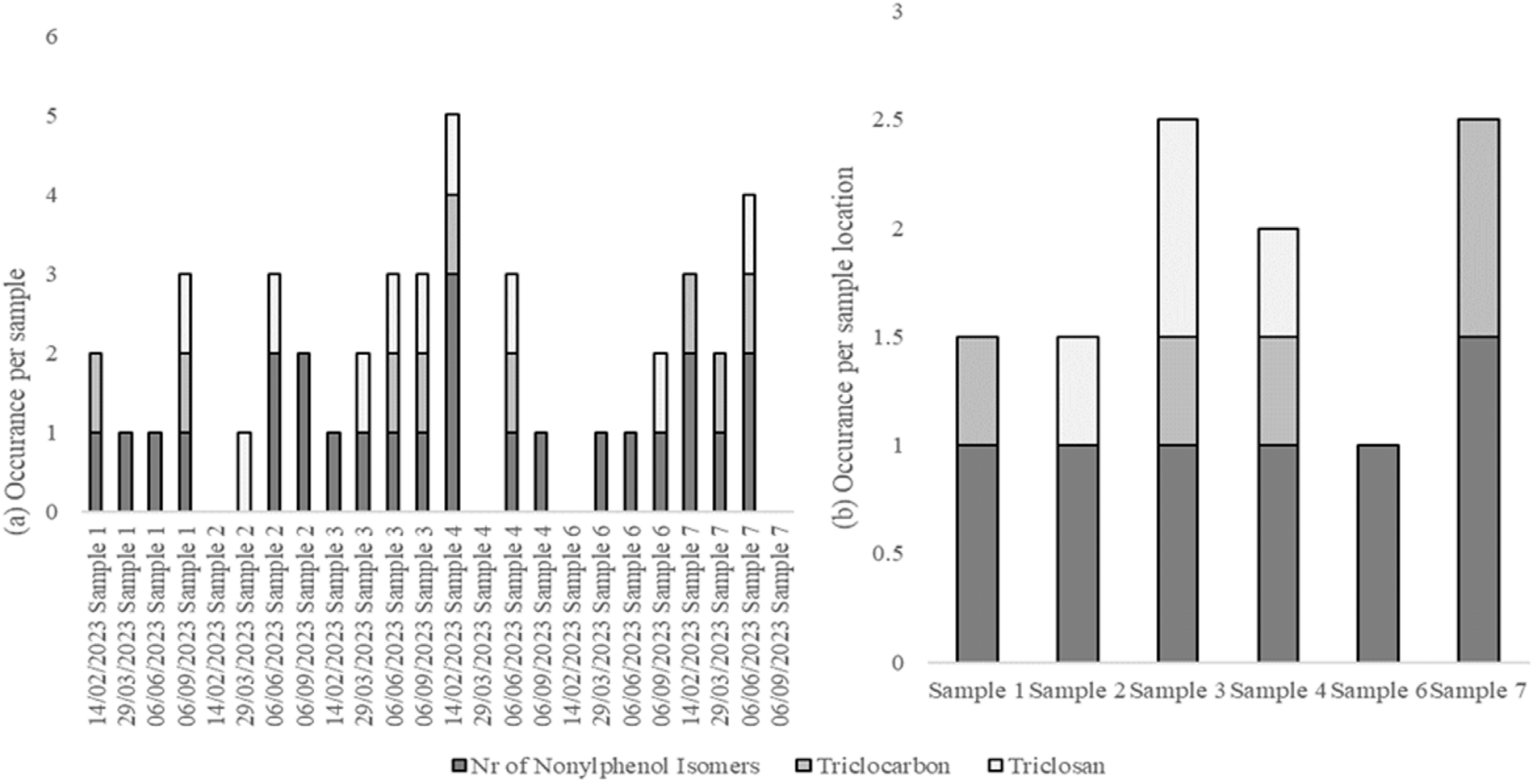
Occurrence of nonylphenol isomers, triclocarban, and triclosan per water sample **(A)** and per sample location **(B)** from the food-processing facility.

Surfactants were detected in most water samples. The detection of nonylphenol isomers, triclocarban, and triclosan is shown in Figure 2.

**FIGURE 3:**
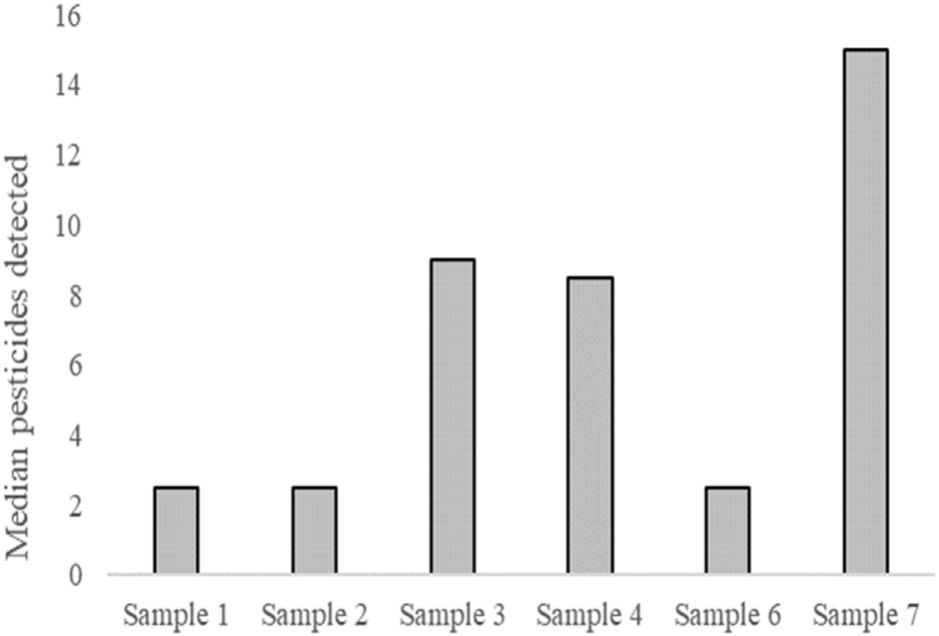
Median number of pesticides detected per sample.

Pesticides were detected in all the samples, according to LC-MS/MS and GC-MS/MS. The median was used to summarize the pesticides detected during screening (Table 4) to illustrate the median number of pesticides per sample (Figure 3). The most pesticides were detected in sample 7.

**TABLE 4:** Pesticides positively identified through LC-MS/MS and GC-MS/MS in various water samples.

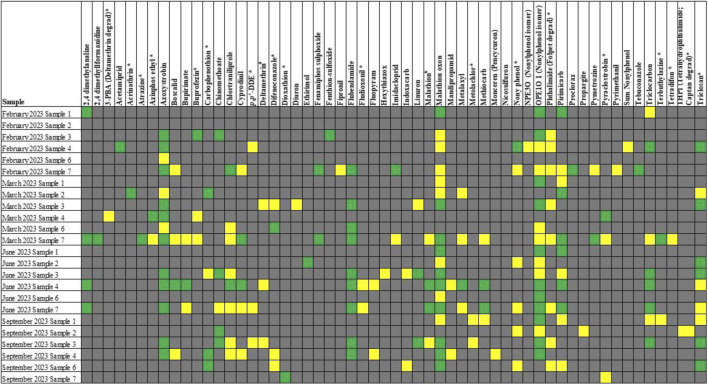

a GC compounds; #, GC and LC compounds; gray: not detected (ND); yellow: limit of detection (DL); green: > limit of quantification (LOQ).

## 4 Discussion and conclusion

*In vitro* bioassays and chemical screening can be used to assess the contribution of food-processing facilities to the quality of wastewater released into the environment. This study used YES and MDA-kb2 assays to assess two effect-based method endpoints, namely, estrogenic and (anti-)androgenic activity. The study revealed that wastewater from a minimally processed vegetable facility contained detergents, disinfectants, and pesticides, displaying considerable hormonal activity, notably estrogenic activity.

### 4.1 YES assay

Yeast-based bioassays are preferable over mammalian bioassays as they lack endogenous receptors that can interfere with cellular response, are simple to conduct, are cheaper, do not require steroid-free media, and are very robust compared with mammalian cells, and therefore, they are ideal for testing extracts from dirty sample matrices (Leusch et al., 2010; Connolly et al., 2011). However, yeast-based bioassays are notably less sensitive than human cell line bioassays (Leusch et al., 2010; Connolly et al., 2011). In this study, sample point 1, the control, was the only point below the DL for all four events, indicating that activities in the facility may have contributed to estrogenic activity. Sample 2 (utensil washbasins) was the only sample where estrogenic activity could be quantified for all sampling events, with EEq values ranging from 3 to 294 ng/L. Compared with the samples from wash basins (average EEq: 146 ng/L), the drain water had much lower activity (average EEq: 0.23 ng/L), possibly due to a dilution effect in the drains. The high variability in estrogenic activity from samples 4, 6, and 7 can be expected as the food-processing activities may vary according to the type of vegetable being processed on a specific day. The proposed ecological effect-based trigger value for estrogenic activity in wastewater is 0.2–0.4 ng/L EEq (Jarošová et al., 2014). Samples 2, 4, 6, and 7 all exceeded this value. However, sample 3 would be more representative of the levels that would be released into the environment. The EEq for sample 3 was 0.23 ng/L, which is at the trigger value level. We recommend monitoring this sample point to ensure that the levels do not increase further.

Disinfectants are added to washing baths and surface-cleaning water to reduce the risk of microbial contamination (van Dijk et al., 2022). Therefore, the cytotoxicity observed in some of the samples was expected in this study. A dilution series should be performed to assess estrogenic activity, and only nontoxic concentrations should be used to calculate the EEqs; however, in toxic samples, the estrogenic activity could be underestimated because of the dilution effect.

### 4.2 MDA-kb2 reporter gene assay

None of the samples showed antiandrogenic activity. Only sample 7, collected in March, tested positive for androgenic activity. The androgenic activity could be from residues (pesticide or organic) present on the produce crates. As with the YES assay, the cytotoxicity observed was expected.

### 4.3 Chemical analyses

Water samples from the facility contained detergents, disinfectants, and pesticides. Total ion chromatograms revealed that the control sample (sample 1) was less complex than the other water samples. This finding supports the results of the YES assay, where only sample 1 was consistently below the assay DL across all sampling events. These findings suggest that food-processing activities can potentially affect the water quality inside facilities. This water is eventually released into the environment via drains. Similar to the YES results, the drains were slightly less complex than the basins, which might be attributed to a dilution effect.

Nonylphenol isomers were detected in almost all of the water samples. Similarly, Biragova et al. (2020) demonstrated that surfactants can be present in wastewater produced by food-processing facilities. Additionally, the screening results indicated that 4-octylphenol was detected in most of the samples; however, this was based on mass spectra and not confirmed with an authentic standard. Beresford et al. (2000) demonstrated that octylphenol can leach across a YES assay plate. The nonylphenol and octylphenol present in the samples may, therefore, be responsible for the leaching observed in the YES plates in this study.

Pesticides were detected in all the samples, with sample 7 showing the highest number of pesticides. Pesticide residues are likely to have been present on fruits and vegetables (Mutengwe et al., 2016) and are likely to have entered the water after processing. Cyprodinil, a fungicide effective against a broad spectrum of plant pathogens^1^, was detected in samples 4 and 7. Go et al. (2015) reported that cyprodinil exerts disruptive effects via an estrogen-dependent pathway. Flubendiamide, a petrochemical pesticide detected in samples 3, 4, 6, and 7, also acts as an estrogen receptor agonist^2^. Triclocarban, a bacteriocide detected in samples 3, 4, and 7, also exhibits estrogen-agonist properties (Iacopetta et al., 2021). The detected pesticides and disinfectants may explain the estrogenic activity observed in this study.

Even at concentrations ranging from as low as a few ng/L, EDCs may cause endocrine disruption in many species, including abnormal vitellogenin induction, altered sex determination, decreased growth rates, delayed reproduction and altered behaviors in fish, and metabolic and reproductive disorders in humans (Ismanto et al., 2022).

### 4.4 Limitations

One notable limitation to the study is its focus on a single minimally processed vegetable facility, which may limit the generalizability of the findings to other food-processing facilities with different operational scales or processes. Additionally, although the study used comprehensive chemical screening and *in vitro* bioassays, it did not incorporate *in vivo* testing, which might provide further insight into the ecological and health impacts of the detected contaminants.

### 4.5 Conclusion

These findings showed that wastewater from a minimally processed vegetable facility contained detergents, disinfectants, and pesticides and displayed hormonal activity (estrogenic and androgenic). The pesticides and disinfectants used in the facility may have contributed to the detected endocrine-disrupting activity. Food-processing facilities produce large volumes of wastewater, and although conventional water quality monitoring typically includes parameters such as conductivity, acidity, turbidity, and the presence of *Enterobacteriaceae*, we recommend expanding monitoring efforts to include endocrine activity and disinfectant byproducts.

## Data Availability

The raw data supporting the conclusion of this article will be made available by the authors, without undue reservation.

## Footnotes

1
https://pubchem.ncbi.nlm.nih.gov/compound/Cyprodinil

2
https://pubchem.ncbi.nlm.nih.gov/compound/11193251#section=BioAssay-Results
